# Effect of the storage atmosphere on metabolomics of harvested tomatoes (*Solanum lycopersicum* L.)

**DOI:** 10.1002/fsn3.923

**Published:** 2019-01-28

**Authors:** Yuma Yokota, Takashi Akihiro, Surina Boerzhijin, Takeshi Yamada, Yoshio Makino

**Affiliations:** ^1^ Graduate School of Agricultural and Life Sciences The University of Tokyo Bunkyo‐ku Tokyo Japan; ^2^ Faculty of Life and Environmental Science Shimane University Matsue City Shimane Japan; ^3^ Department of P‐plus Project Sumitomo Bakelite Co. Ltd. Shinagawa‐ku Tokyo Japan

**Keywords:** environmental gas composition, metabolomics, modified atmosphere packaging, postharvest storage, principal component analysis, tomato

## Abstract

Harvested tomatoes were stored under atmospheres that were normoxic, anoxic, or modified (altered O_2_ and CO_2_ concentrations). Each atmosphere was created by storing the tomatoes at 25°C for up to 8 days in different kinds of pouches. During storage, metabolites of the tomatoes were measured using metabolomics. We obtained score plots of the metabolites on eighth day of storage by principal component analysis. There was a tendency for groups to be divided on the basis of score plot according to the composition of each gas. PC1 and PC2 seemed to correspond to the influence of O_2_ and CO_2_ concentrations, respectively, and the total contribution rate of the two axes was 72%, so that we concluded that the metabolites were affected mainly by O_2_ and CO_2_ concentrations. The results indicate that metabolomics may be an effective tool to reveal the relationship between metabolic state of harvested fruits and the atmosphere.

## 1 INTRODUCTION

Modified atmosphere packaging (MAP) is a promising method for maintaining the freshness of harvested fruits and vegetables by sealing them in a pouch where gas permeability can be controlled (Kader, Zagory, Kerbel, & Wang, 1989). The effectiveness of this method has been demonstrated in terms of retention of the green color and antioxidative effect of broccoli heads (Serrano, Martinez‐Romero, Guillén, Castillo, & Valero, 2006), maintenance of the hardness and color of persimmons (Cia et al., 2006), and decrease in the microbial rotting of tomatoes (Nakhasi, Schlimme, & Solomos, 1991), among others. These effects are observed under in‐package atmospheres, including low O_2_ and high CO_2_ concentrations created by the interaction of the controlled gas permeability of the packaging pouch and respiration by fruits and vegetables (Kader et al., 1989). Furthermore, there have been reports of dynamic changes in the functional ingredients of vegetable tissue during storage. Makino, Soga, Oshita, Kawagoe, and Tanaka (2008), Deewatthanawong, Rowell, and Watkins (2010), and Mae et al. (2012) all reported that atmospheres combining low O_2_ and high CO_2_ concentrations were effective at increasing γ‐aminobutyric acid (GABA) concentration in tomatoes. Makino, Nishimura, Oshita, Mizosoe, and Akihiro (2018) reported that sulforaphane concentration in broccoli florets also increased under atmospheres combining low O_2_ and high CO_2_ concentrations. These results suggest that the gas composition of the storage atmosphere may affect the metabolism in fruits and vegetables.

Metabolomics has been established as a research tool to comprehensively investigate metabolite concentrations in biological systems and has been used to analyze molecular phenotypes (Fiehn, 2002; Van der Werf, 2005; Weckwerth & Morgenthal, 2005). Research into metabolomics in the field of plant science has been mainly conducted using *Arabidopsis thaliana*, as reported by Hanada et al. (2010). There are few cases where metabolomics has been applied to the field of postharvest technology. Pedreschi et al. (2014) reported that metabolomics was effective for investigating the ripening mechanism of avocado fruit, while Hatoum, Annaratone, Hertog, Geeraerd, and Nicolai (2014) used metabolomics to characterize the influence of the use of chemicals (calcium, potassium, and triazole fungicides) on primary metabolites in Braeburn apples. Pedreschi et al. (2009) were able to identify the cause of core breakdown in Conference pears by metabolic profiling under low O_2_ or high CO_2_ conditions.

Ripening of tomatoes involves dramatic metabolic fluctuations (Adams‐Phillips, Barry, & Giovannoni, 2004; Carrari, Asis, & Fernie, 2007; Giovannoni, 2001, 2004; Rose, Saladié, & Catalá, 2004), and the metabolism of the stored tomato fruit is known to be affected by the ambient gas composition (Mae et al., 2012). The purpose of this study was to investigate the effects of the gas composition of the atmosphere during postharvest storage on the properties of the tomato by carrying out a comprehensive analysis of the metabolic state of the tomatoes with the use of metabolomics.

## 2 MATERIALS AND METHODS

### 2.1 Plant materials

We used tomatoes (*Solanum lycopersicum* L.) cv. F_1_ hybrid Momotaro, the dominant fresh‐market tomato grown in Japan, at the breaker stage (red color first becomes noticeable) harvested on January 31, 2017, at a glasshouse in Kochi Prefecture, Japan.

### 2.2 Packaging materials

We used two kinds of microperforated pouches made from polypropylene (surface area: 0.085 m^2^, thickness: 0.025 mm; Sumitomo Bakelite Co., Ltd., Tokyo, Japan) with 1.66 × 10^6^ (normoxic atmosphere) or 9.0 × 10^3^ ml m^−2^ day^−1^ atm^−1^ (modified atmosphere, MA) O_2_ transmission rates. The diameter of the perforation was so small that water vapor could hardly pass. Each MA pouch also contained 10 g CO_2_‐absorbent (Ageless C^®^; Mitsubishi Gas Chemical Co, Inc., Tokyo, Japan) because an atmosphere containing 0% CO_2_ is suitable for storing tomatoes (Dilley, 1978). The third pouch used a high‐barrier material, with an 8.0 ml m^−2^ d^−1^ atm^−1^ O_2_ transmission rate (anoxic atmosphere) made from laminated film (nylon/polyethylene; surface area: 0.0936 m^2^, thickness: 0.118 mm; Lamizip^®^; AS ONE Co., Ltd., Osaka, Japan).

### 2.3 Measurement of in‐pouch gas composition, and the physicochemical properties and the metabolite concentrations of the stored tomatoes

Two tomatoes were sealed in each pouch, and a total of 12 pouches of each type were stored at 25°C for up to 8 days. Every 2 days, three replicate pouches of each type were selected at random, and the atmosphere and tomatoes in these replicate pouches were sampled (six tomatoes which were not stored were sampled as 0‐day tomatoes on the day of the start of the storage). We measured the O_2_ and CO_2_ concentrations in the pouches containing tomatoes using a gas analyzer (CheckMate 3, Dansensor A/S, Ringsted, Denmark). Immediately after analyzing the gas concentrations, the six tomatoes from the three replicate pouches of each treatment were sampled, and their fresh masses were measured. We calculated mass retention from the measured mass value using Equation (1):

(1) $$M_{r}=\frac{100\cdot M_{t}}{M_{0}}$$

where *M* is the mass of a tomato (g), subscript *r* denotes the retention, *t* stands for an arbitrary time, and 0 stands for the initial day.

We measured Commission Internationale de l’Éclairage (1976) L*a*b* color space values using a colorimeter (CM‐700 d; Konica Minolta Japan Inc., Tokyo, Japan). It is known that the color space value a*/b* is one of the indicators used for determining the ripeness of tomatoes based on the pericarp color, and this value increases with ripening (Arias, Lee, Logendra, & Janes, 2000). We milled each whole tomato in liquid nitrogen using a grind mixer (GM200; Verder Scientific GmbH & Co. KG, Haan, Germany) shortly after measuring the mass and color space values. A 30 mg sample of frozen powder obtained after liquid nitrogen treatment was used for the measurement of metabolite concentrations. Sample pretreatment was conducted according to the method reported by Pongsuwan et al. (2007). An aliquot (50 μl) of methoxamine (20 mg/ml pyridine) was added to the tomato preparation and incubated at 30°C for 90 min, after which 25 μl N‐methyl‐N‐(trimethylsilyl)trifluoroacetamide was added, and the solution was incubated at 37°C for 30 min. GC/MS analysis was conducted using a GCMS‐QP2010 Ultra (Shimadzu, Tokyo, Japan), and simultaneous analysis of 475 metabolites was carried out using the Smart Metabolite Database v. 2.0 (Shimadzu, Tokyo, Japan). This analysis used a DB‐5 column (30 m × 0.25 mm i.d.; film thickness: 1.00 μm; J&W Scientific, Folsom, CA, USA). The GC column temperature was programmed to increase to 100°C (0–1 min), from 100 to 320°C (5–25 min, 22°C/min) and maintained at 320°C (25–35 min), so that the total GC run time was 60 min. The injection volume was 1 μl in the splitless mode, and the mass spectrometry conditions were set as follows: ionization voltage, 70 eV; ion source temperature, 200°C; interface temperature, 280°C; and full scan mode, range of 35–600 *m*/*z* and scan velocity 0.20 scans/s. The concentration of a metabolite was expressed as a specific value against the concentration of the internal standard (ribitol). We normalized the distribution of each specific metabolite concentration in the range of 0–1 using Eq. (2):

(2) $$C_{s}=\frac{C_{i}-C_{\min}}{C_{\max}-C_{\min}}$$

where *C* is the specific concentration of the compound, subscript *i* stands for an arbitrary value, *s* represents the standardized value, max stands for the maximum, and min stands for the minimum value.

### 2.4 Statistical analysis

To analyze the mean data from the different treatments for the mass retention and a*/b* parameters, ANOVA was carried out. Where significant, we carried out multiple pairwise comparisons using Tukey's honestly significant difference test and JMP^®^ Pro v. 13.2.0 (SAS Institute Inc., Cary, NC, USA).

We carried out principal component analysis (PCA) using The Unscrambler X v. 10.3 (CAMO software Japan, Tokyo, Japan), with the time course of metabolite concentration as an input variable.

## 3 RESULTS AND DISCUSSION

### 3.1 Changes in in‐pouch atmospheres over time

**Figure 1:**
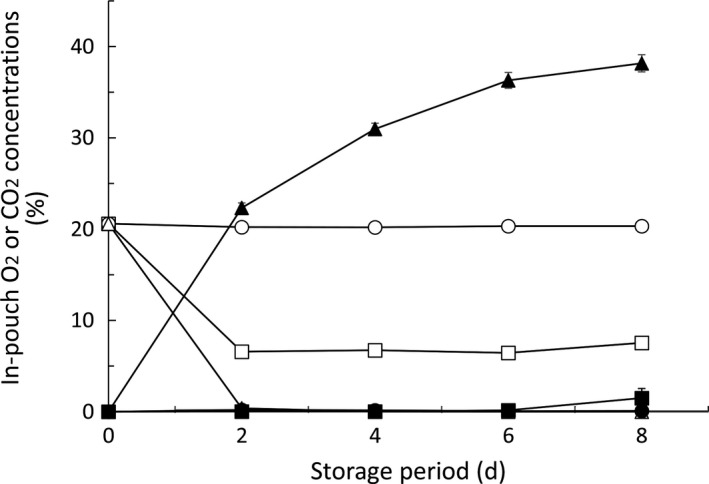
Changes in in‐pouch atmosphere over time. Circles, triangles, and squares represent normoxic, anoxic, and modified atmospheres, respectively. Open and closed symbols stand for O_2_ and CO_2_ concentrations, respectively. Values are the means ± SE of three observations from three different biological samples (replicates). On occasions, the error bar was smarter than the symbol

Figure 1 shows the changes in O_2_ and CO_2_ concentrations in the different types of pouches containing tomatoes. The O_2_ and CO_2_ concentrations in the low‐barrier microperforated pouches with the 1.66 × 10^6^ ml m^−2^ d^−1^ atm^−1^ O_2_ transmission rate (normoxic pouch) were similar to the ambient air due to the high permeability of the pouches. The O_2_ concentration in the high‐barrier pouches (anoxic pouches) decreased to almost 0% at 2 days, and the CO_2_ concentration continued to rise after 2 days. The O_2_ concentration in the microperforated pouches with the 9.0 × 10^3^ ml m^−2^ d^−1^ atm^−1^ O_2_ transmission rate (MA pouch) was maintained at *ca*. 7% after 2 days, while CO_2_ concentration was maintained at about 0% during the storage period due to the presence of the CO_2_ absorbent (Figure 1).

### 3.2 Changes in tomato mass retention over time

**Figure 2:**
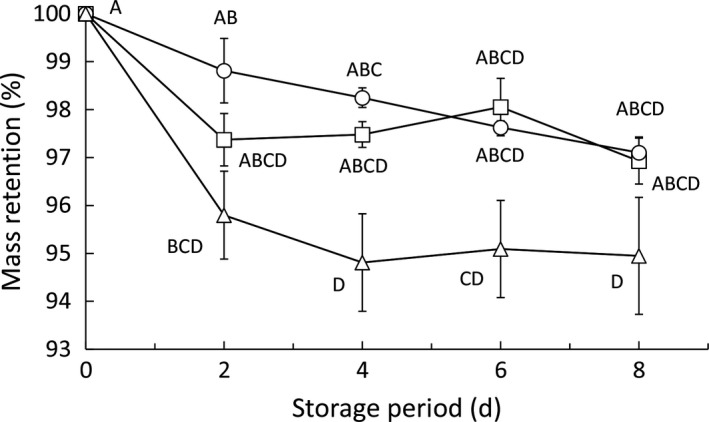
Changes in mass retention over time. Circles, triangles, and squares represent normoxic, anoxic, and modified atmospheres, respectively. Values are the means ± SE of observations from six different biological samples (replicates). Symbols followed by a common letter within the same figure indicate that there were no significant differences (*p *>* *0.05, Tukey's honestly significant difference test). On occasions, the error bar was smarter than the symbol

Figure 2 shows the changes in mass retention of the tomatoes over time (mean mass of the tomatoes at zero days was 362 g per pouch). There was no significant difference between the mass retention of the 0‐day tomatoes, and those of the tomatoes stored under normoxic or MA conditions. In contrast, the mass retention of the tomatoes stored for 2 days under anoxic conditions was significantly lower than the tomatoes at zero days (Figure 2). This is probably because polypropylene was used as the packaging material for the normoxic and MA pouches, whereas nylon/polyethylene, with a greater moisture permeability than polypropylene (Zeman & Kubík, 2007), was used for the anoxic pouch, resulting in greater moisture loss and, hence, decreased mass retention in the stored tomatoes. It has been reported that storage under an anoxic environment can cause carbon dioxide injury in tomatoes (Yang & Chinnan, 1987). However, the cause of the rapid weight loss of tomatoes stored under anoxic conditions is not water leakage due to the injury. We confirmed that damages such as fruit cracking did not occur in those tomatoes. This may be because the storage period was relatively short.

### 3.3 Changes in color space value over time

**Figure 3:**
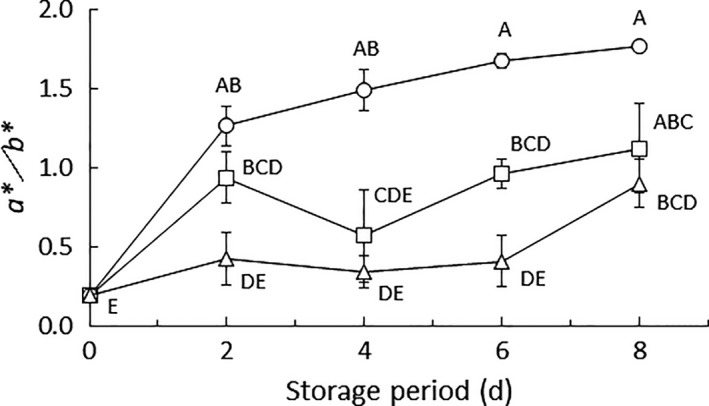
Changes in color space value over time. Circles, triangles, and squares represent normoxic, anoxic, and modified atmospheres, respectively. Values are the means ± SE of observations from six different biological samples (replicates). Symbols followed by a common letter within the same figure indicate that there were no significant differences (*p *>* *0.05, Tukey's honestly significant difference test). On occasions, the error bar was smarter than the symbol

Figure 3 shows the changes in a*/b* of the tomatoes over time. The tomatoes stored under normoxic or MA conditions exhibited a*/b* values at 2 days of storage and later, which were significantly higher than those at zero days (except for MA at 4 days). This indicates that the ripening of tomatoes began under the normoxic or MA conditions on and after 2 days. Regarding the tomatoes stored under MA or anoxic conditions, a*/b* was significantly lower than for the tomatoes stored under normoxic conditions (for MA, these differences were significant only at 4 and 6 days; Figure 3). These results suggest that ripening was inhibited under the MA or anoxic conditions. This may be because the respiration rate of tomatoes under normoxia was higher than that under the other atmospheric conditions.

This result suggests that the low O_2_ environment under the MA or anoxic conditions suppressed pericarp color development associated with tomato ripening and possibly preserved the freshness of the tomato fruits. However, as mentioned above, long‐term storage under an anoxic environment can severely injure the tomatoes (Yang & Chinnan, 1987). Therefore, MA conditions are more suitable for storing tomatoes than anoxic conditions.

### 3.4 Effects of atmosphere gas composition during storage on changes in metabolite concentrations of tomatoes

**Figure 4:**
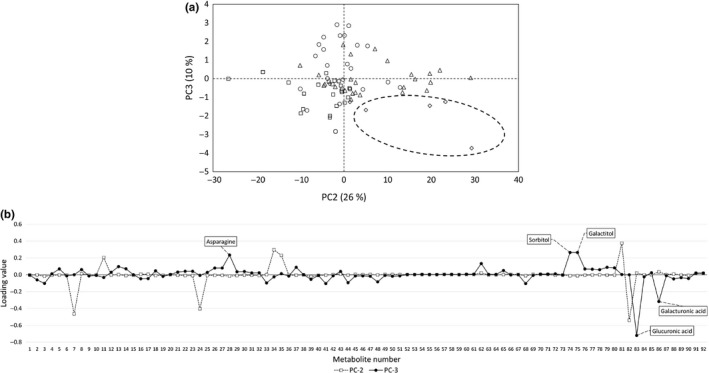
(a) Score plot of metabolite concentrations in tomatoes (incorporating all data from 0 to 8 days). Circles, triangles, and squares represent normoxic, anoxic, and modified atmospheres, respectively. Diamonds stand for zero days and are delimited by a broken‐line circle. (b) Absolute loading values of Figure 4a

**Figure 5:**
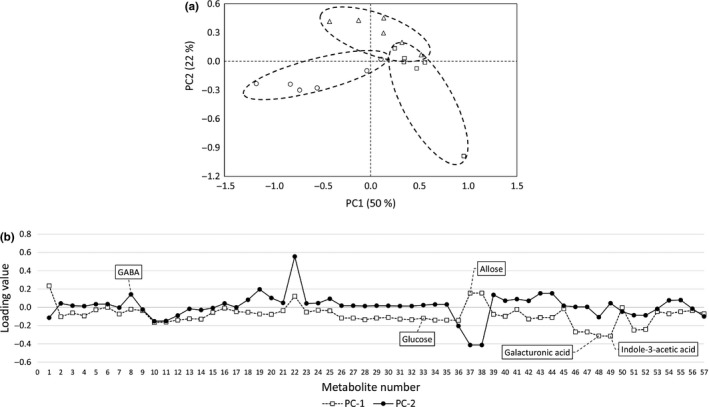
(a) Score plot of metabolite concentrations in tomatoes (incorporates data from only 8 days). Circles, triangles, and squares represent normoxic, anoxic, and modified atmospheres, respectively. (b) Absolute loading values of Figure 5a

We obtained score plots by PCA, using metabolite concentrations in the tomatoes (Figure 4). According to the results in Figure 4a, PC3 may be associated with the storage period because scores at zero days were lower than the scores at later storage dates. According to the results of loading (Figure 4b), the absolute values of glucuronic acid, galacturonic acid, asparagine, sorbitol, and galactitol concentration were higher than those of other metabolites. These metabolites may be associated with the duration of the storage period. However, clear grouping was not observed in the results of the score plots, which incorporated all the data from 0 to 8 days of storage (Figure 4a), possibly because metabolite concentrations were affected not only by storage atmosphere but also by storage period (Mae et al., 2012). Therefore, we used a score plot from the data from only 8 days, which are thought to be those most strongly affected by storage atmosphere (Figure 5). According to the results in Figure 5a, there was a tendency for the groups to be distinguished according to the prevailing gas composition during storage. Because scores of PC2 tended to be elevated under anoxic conditions, PC2 appears to reflect the influence of environmental CO_2_ concentrations. According to the results of loading (Figure 5b), the absolute value of GABA was higher than that for other metabolites. Deewatthanawong et al. (2010) reported that atmospheres that included high CO_2_ concentrations were effective at increasing GABA in tomatoes. This finding may support the results observed in the present study.

In contrast, PC1 seems to reflect the influence of O_2_ concentrations. According to the results of loading (Figure 5b), the absolute concentration values of many kinds of sugars (glucose, allose, etc.) were higher than those for other metabolites. This suggests that the atmospheric O_2_ level affects the production of many kinds of sugars. The absolute loading value of galacturonic acid was also higher than that for other metabolites. This compound is the product of pectin decomposition (Daas, Meyer‐Hansen, Schols, De Ruiter, & Voragen, 1999), and this result may indicate that decomposition of pectin (associated with the ripening‐induced softening of tomato fruits) is depressed under low‐O_2_ conditions. Furthermore, the absolute loading value of indole‐3‐acetic acid was higher than that for other metabolites. It has been reported that this compound stimulates the production of 1‐aminocyclopropanecarboxylic acid (ACC), the precursor of ethylene (Adams & Yang, 1979), which is the gaseous plant hormone that stimulates senescence and fruit ripening. The result in Figure 5 may indicates that the production of ACC is depressed under low‐O_2_ conditions, supporting the result that color development, stimulated by ethylene, was inhibited under low‐O_2_ conditions (Figure 3). The contribution rates of PC1 and PC2 were 50% and 22%, respectively, resulting in a total explanation of 72% of the total variance. Therefore, the metabolite concentrations were mainly affected by environmental O_2_ and CO_2_ concentrations. According to the results mentioned above, metabolomics appeared to be an effective tool by which to reveal the relationship between the metabolic state of harvested tomatoes and the atmosphere during storage.

## CONFLICT OF INTEREST

We have the following interests: Takeshi Yamada is employed by Sumitomo Bakelite Co. Ltd. There are no patents, products in development or marketed products to declare. This does not alter our adherence to all the Food Science and Nutrition policies on sharing data and materials in the instructions to authors.

## ETHICAL STATEMENTS

This study does not involve any human or animal testing.
